# To value the model of psychiatric hospital admission, from 2013 to 2017 in local health service in a 240.000 people area of northern Italy

**DOI:** 10.1192/j.eurpsy.2021.993

**Published:** 2021-08-13

**Authors:** M. Giacomin

**Affiliations:** Mental Health, AUSL 2 Veneto Italy, JESOLO (VE), Italy

**Keywords:** Trends in Psychiatric Patients Admissions, Admission, Patients, epidemiology

## Abstract

**Introduction:**

Psychiatric Patients Admissions in Mental health Service of Treviso (Italy) were compared from 2013 to 2017. Trends of Admissions take onto consideration, the presence of Menthal Health Service for Outpatients Care.

**Objectives:**

To point out the distribution of Diagnosis made in Different Years for different patients ages.

**Methods:**

For every patient has been considered the following date : Sex, Age, Marital State, Profession, Psychiatric Diagnosis, Days of Admission, Geografic Origin and KInd of Admission (Voluntary / Involuntay).

**Results:**

It is noticeable the different percentage of Psychiatric Diagnosis in 2013 rather than in 2017. In 2017 it happened a more amount of Psychiatric Admission of Subjects with Substance Addiction Related Disturbs (Alcool included) and Atypical Depression Sindrome and Borderline and Cluster B Perrsonalòity Disorders. Lower amount instead was verified for Diagnosis of Schizofernia, Neurosis and Oligofrenia. Beside it was noticed, an earlier onset of Psychotic Sindrome in Young people often related with Substance Abuse. In the 2017 besides was lower the amount of Involuntary Admission (T.S.O. in Italy) compared with 2013.

**Conclusions:**

Different distribution of Diagnosis is explained by the Evolution Diagnosis Orientation (from D.S.M. IV to I.C.D. 10) About the increased Diagnosis of Substance Addiction Disturbs and Younger age of same subjescts seems caused by a different treatment’s Strategy with brief selective Admissions. Furthermore lesser Involonary Admission seems due to best knowledge of every patients. The most of Theese were indeed already known by Ambulatory Outpatient Mental Health Service.

